# The Auto-Combustion Method Synthesized Eu_2_O_3-_ ZnO Nanostructured Composites for Electronic and Photocatalytic Applications

**DOI:** 10.3390/ma15093257

**Published:** 2022-05-01

**Authors:** Thekrayat H. AlAbdulaal, Vanga Ganesh, Manal AlShadidi, Mai S. A. Hussien, Abdelfatteh Bouzidi, Hamed Algarni, Heba Y. Zahran, Mohamed Sh. Abdel-wahab, Ibrahim S. Yahia, Samia Nasr

**Affiliations:** 1 Laboratory of Nano-Smart Materials for Science and Technology (LNSMST), Department of Physics, Faculty of Science, King Khalid University, P.O. Box 9004, Abha 61413, Saudi Arabia; talabdulaal@kku.edu.sa (T.H.A.); miss.memaa_-_@hotmail.com (M.A.); halgarni@kku.edu.sa (H.A.); dr_hyzahran@kku.edu.sa (H.Y.Z.); 2 Department of Chemistry, Faculty of Education, Ain Shams University, Roxy, Cairo 11757, Egypt; maisalehamar@gmail.com; 3 Nanoscience Laboratory for Environmental and Bio-Medical Applications (NLEBA), Semiconductor Laboratory, Metallurgical Lab, Department of Physics, Faculty of Education, Ain Shams University, Roxy, Cairo 11757, Egypt; 4 Research Unit, Physics of Insulating and Semi-insulating Materials, Faculty of Sciences, University of Sfax, B.P. 1171, Sfax 3000, Tunisia; abdelfatteh_bouzidi83@yahoo.fr; 5 Preparatory Year Program, Shaqra University, Al-Quwayiyah Branch, Sahqra 19248, Saudi Arabia; 6 Materials Science and Nanotechnology Department, Faculty of Postgraduate Studies for Advanced Sciences, Beni-Suef University, Beni-Suef 62511, Egypt; mshaabancnt@gmail.com; 7 Research Center for Advanced Materials Science (RCAMS), King Khalid University, P.O. Box 9004, Abha 61413, Saudi Arabia; 8 Department of Chemistry, Faculty of Sciences and Arts Touhama, King Khaled University, Muhayil Asir 63311, Saudi Arabia; smohmed@kku.edu.sa; 9 Electrochemistry, Materials, and Environment, Preparatory Institute for Engineering Studies, Kairouan 3100, Tunisia

**Keywords:** combustion method, Eu_2_O_3_-ZnO nanostructured composites, structural, optical, electrical, photocatalytic activity

## Abstract

An efficient and environmentally friendly combustion technique was employed to produce ZnO nanopowders with different Eu concentrations (from 0.001 g to 5 g). The structural morphology of the Eu_2_O_3_-ZnO nanocomposites was examined using XRD, SEM, and infrared spectroscopy (FT-IR). In addition, UV-Vis diffuse reflectance spectroscopy was also used to investigate the effects of europium (Eu) dopant on the optical behaviors and energy bandgaps of nano-complex oxides. The photocatalytic degradation efficiency of phenol and methylene blue was investigated using all the prepared Eu_2_O_3_-ZnO nanostructured samples. Photocatalytic effectiveness increased when europium (Eu) doping ratios increased. After adding moderate Eu, more hydroxyl radicals were generated over ZnO. The best photocatalyst for phenol degradation was 1 percent Eu_2_O_3_-ZnO, while it was 0.5 percent Eu_2_O_3_-ZnO for methylene blue solutions. The obtained Eu_2_O_3_-doped ZnO nanostructured materials are considered innovative, promising candidates for a wide range of nano-applications, including biomedical and photocatalytic degradation of organic dyes and phenol.

## 1. Introduction

Recently, more attention has been paid to wastewater treatment for human society. Phenolics are particular contaminants frequently produced from a wide range of industrial products, including textiles, pesticides, gunpowder, dyes, and plastics [1,2]. Most of these compounds cause severe damage in various natural areas, in which phenols are difficult to remove using traditional organic treatment techniques due to their high stability and toxicity [3,4]. ZnO exhibits remarkable properties, including high electrochemical coupling, high chemical stability, low dielectric constant, and high exciton binding energy of about 60 meV. It also shows a broad bandgap (approximately 3.37 eV at room temperature), sufficient binding, thermal conductivity, and UV protection [5,6]. ZnO films are also thought to be high-transparency materials, with refractive indices ranging from infrared (IR) to visible. As a result, many ZnO-based materials are widely used in different technical products, such as UV light emitters, windows for solar cells, thin-film transistors, biological and chemical gas sensors, biomedical gas sensors, and varistors [7]. Among these properties, ZnO has also been an excellent candidate for photocatalyst applications in wastewater treatment due to its highly photocatalytic characteristic with ultraviolet (UV) light [8,9,10]. Several synthesis methods have been studied to increase ZnO photocatalytic properties, including sol-gel, solid-state reaction, and solvothermal [11,12,13]. In addition, different non-metal or metal dopants have been doped into a pure ZnO matrix to improve its optical, chemical, and physical properties [14,15,16].

When pure ZnO is doped with different rare earth elements such as La^3+^, Nd^3+^, Dy^3+^, Sm^3+^, and Ce^3+^, it increases the efficiency of photocatalytic studies [17,18]. Consequently, ZnO doped with europium offers a practical approach to photocatalytic behavior since electron trapping is significantly effective through Eu^2+^ and Eu^3+^ valence sites [19]. Yang and Yu et al. [20,21] prepared to enhance the photocatalytic effects of hydrothermal synthesized Eu^3+^-ZnO nanostructured materials. Additionally, Phuruangrat et al. applied the sonochemical approach to manufacture Eu-doped ZnO nanomaterials to investigate structure morphology and photocatalytic behaviors. It was shown that the dopant with 3 mol% Eu had the highest efficiency of photocatalytic activities [22]. Importantly, ZnO-Eu nanostructures have been designed to promote photocatalytic activity, display transparent coatings, and improve UV emissions from photoluminescence (PL) [23,24]. In addition, several studies have considered europium (Eu) rare earth elements to be utilized to enhance the optical parameters of ZnO [25]. Europium compounds are also utilized in computer monitoring, neutron scintillators, X-ray screens, mercury vapor lamps with significant intensity, detectors for charged parts, and optically read memory systems [26,27]. In addition to these properties, there are some challenges involved in preparing Eu_2_O_3_-doped ZnO nanostructures. Europium is the rarest and most expensive rare earth element (REE), representing only 0.05–0.1% of REEs in inorganic monazite. The most significant behavior of the Eu element is its luminescence, which can be described by way of light emissions, which are, significantly the energy absorption of the material [27]. This behavior controls the primary Eu usage in various potential applications, such as phosphorus, emitting colored light via electroluminescent procedures, and computer and television displays. In addition, many scientists have utilized luminescence spectroscopy based on europium ions to elucidate the structure of natural and synthetic compounds [27]. Although some reports are available on photocatalytic studies of Eu_2_O_3_-doped ZnO nanostructures [20,21,22], there is still a lack of systematic studies on the structural, morphological, spectral, and photodegradation properties of Eu_2_O_3_-doped ZnO nanostructures. Nonetheless, based on the most special properties of Eu_2_O_3_, the present study used the combustion method to prepare Eu_2_O_3_-ZnO nanostructures for enhancing photocatalytic properties. 

Further, X-ray diffraction (XRD) and scanning electron microscopy (SEM) were used to characterize the structural morphology of the Eu_2_O_3_-doped ZnO materials. The improvements of ZnO optical characteristics with europium (Eu) were investigated using UV-Vis diffuse reflectance spectroscopy (DR) and Fourier transformation infrared spectroscopy (FT-IR) at varying concentrations ranging from 0.001 g to 5 g. Finally, the photocatalytic performance of visible photodegradation of ZnO and Eu_2_O_3_-doped ZnO was estimated using Ph and MB concerning illumination time.

## 2. Experimental Techniques

### 2.1. Material Growth

In this work, we synthesized ZnO nanoparticles (NPs) doped with europium rare earth metal (Eu_2_O_3_) using a combustion process. Initially, 5 g of Zn(NO_3_)_2_·6H_2_O was mixed and well ground in ceramic crucibles with one gram of gum acacia. Next, eight concentrations of europium (III) nitrate elements ranging from 0.001 g to 5 g were added to the previous mixture, liquified in 5 mL of distilled water, and then kept at 100 °C for 48 h until a completely dried gel formed. After that, the dried gel’s combustion process was applied without further purification at 600 °C for two hours before being cooled at ambient temperature. Gum acacia was used as a fuel in this preparation procedure to help convert the ZnO structure from crystallinity to nanoscale by expanding its elements within the matrix. Table 1 illustrates the abbreviations assigned to the prepared samples as S0 to S7, respectively. 

### 2.2. Devices and Measurements

The crystal structure of the prepared samples was characterized using a Shimadzu LabX-XRD-6000 X-ray diffractometer, Kyoto, Japan, with *CuK_α_* = 1.54 Å radiation in the angle range from 5° to 80°. A JSM-6360 scanning electron microscopy (SEM) with an operating voltage of 20 kV (JEOL, Tokyo, Japan) was used to analyze the morphology of the prepared nanoparticles. Moreover, the ultraviolet-visible spectra of all prepared samples were measured using a UV-3600 UV-Vis spectrophotometer (Shimadzu, Kyoto, Japan), with a wavelength range from 2200 nm to 1600 nm and a step scan of 5 nm. A Thermo Scientific DXR FT-IR Spectrometer was used to study the Fourier transform infrared (FT-IR) spectra of Eu_2_O_3_-doped ZnO nanocomposites in the wavenumber range from 400 to 4000 cm^−1^.

### 2.3. Photocatalytic Measurements

Different types of organic pollutants, such as methylene blue and phenol, were used in a thermostatic photoreactor fitted with a multi-position magnetic stirrer for photocatalytic measurements.

#### 2.3.1. Design of the Ultraviolet-Visible Photoreactor

A wooden photoreactor was used to test the visible photocatalytic operation of all samples under investigation for industrial wastewater treatment under different experimental conditions [28]. The reactor is divided into two portions on the inside and outside. A hardwood frame of 100 cm in height, 95 cm in length, and 65 cm in width makes up the exterior component. In the inner part, seven white (400–700 nm) bulbs and seven blue (18 watts) lamps, each having a spectrum higher than 420 nm, are individually controlled. The wavelength of the visible lamp is larger than 420 nm and for the UVA lamp is 367 nm. The intensity of visible and UVA lamps is 18 watts each. The system is controlled primarily by ON/OFF buttons. The air fan is also attached to the photoreactor for an internal circulating air system to maintain the photoreactor temperature at room temperature. A magnetic stirrer with a multi-position was used to stir the prepared solution.

#### 2.3.2. Photocatalytic Irradiation

The prepared samples of 0.01 g of each doping concentration were dissolved in 100 mL of organic solution, either MB or Ph, with the attention of 20 mg/L. The complete setup was kept in the dark for around 30 min to examine chemisorption until the equilibrium state was obtained. After a 10-min interval of irradiation, 1 mL of the solution sample was withdrawn and centrifuged at 3000 rpm to remove all powder suspension. The remainder of the mixture was exposed to visible light again. The sample activity was measured every 15 min during the irradiation. To investigate the main oxidant species of the photocatalytic reaction, photocatalytic experiments were also carried out on different scavengers. Moreover, following up on the dependency of the photocatalytic performance on light irradiation and its mechanism, trapping experiments with visible-light irradiation for the active species produced during the photodegradation reaction were notably studied. Different agents, including sodium chloride (Cl^−^), sodium nitrate (NO_3_^−^), isopropyl alcohol (IPA), and ascorbic acid (AA), were employed as the scavengers for h^+^, e^−^, ^•^OH, and ^•^O_2_^−^, respectively. Finally, the photodegradation process was detected using a UV-Vis spectrophotometer (200–800 nm). 

The Scherrer equation was employed to investigate the XRD data and the crystallite size (D) of the prepared Eu_2_O_3_-doped ZnO nanocomposites [29,30].

(1) D=0.9λ/βcosθ

Separately, the dislocation density (η), as well as lattice strain (*ε*) of the pure ZnO and Eu_2_O_3_-doped ZnO, were evaluated using the coming expressions [31,32]:

(2) $$\eta =\;\;1\;/D^{2}$$

(3) ε=βcosθ/4

where *λ* is the X-ray wavelength in the unit of nm, *β* is the full width at half maximum (*FWHM*) in terms of radians, and *θ* is the diffraction angle in the degree unit. Dislocations are imperfections in a crystal related to the misregister of the lattice in one part of the crystal concerning another part.

Tauc’s model was used to calculate the optical bandgap (*E_g_*) of pure and Eu_2_O_3_-doped ZnO nanostructured samples using the following equations [29,33]:

(4) $$F(R)=\frac{{{}^{\left(1-R\right)}}^{2}}{2R}=\frac{K}{S}$$

(5) $$\alpha =\frac{F(R)}{t}$$

(6) $${\left(\alpha h\nu \right)}^{1/n}=A^{1/n}(h\nu -E_{g})$$

Here, *F*(*R*) represents the material reflectivity using the Kubelka–Munk model, *R* is reflectance, *K* is the molar absorption coefficient, and *S* is the scattering quantity. In Equations (5) and (6), *α* is the absorption index; *t* is the material thickness; *ν* is the photon frequency; *h* is the Planck constant; *A* is the band tailing factor, with values ranging from 1 × 10^5^ cm^−1^·eV^−1^ to 1 × 106 cm^−1^·eV^−1^; and *E_g_* is a bandgap [34]. In addition, the values of *n* in Equation (6) are either *n* = ½ for direct optical bandgaps or *n* = 2 for indirect optical bandgaps of the studied samples determined by using the following relations:

(7) $${\left(\alpha h\nu \right)}^{2}=A^{2}(h\nu -E_{g})$$

(8) $${\left(\alpha h\nu \right)}^{1/2}=A^{1/2}(h\nu -E_{g})$$

The catalytic efficiency of the prepared samples was estimated from the degradation (%) and the process rate [35]:

*% of degradation* = (*C*_0_ − *C*/*C*_0_) × 100%, (9)

where *C*_0_ is the starting concentration of MB or Ph and *C* is noted as the concentration of the MB or Ph at various times. The photocatalytic degradation reaction is a virtual first-order reaction, as the pollutant’s concentrations are within the millimolar range [35]:

*ln* (*C*/*C*_0_) = *kt*, (10)

where *k* is the constant amount and *t* is the irradiation time in minutes.

The reaction between electrons and the adsorbed H_2_O is illustrated in the following expressions [36,37,38,39,40]:

ZnO + h^+^ → ZnO(e^−^) + ZnO(h^+^), (11)

e^−^ + Eu^3+^ → Eu^2+^, (12)

Eu^2+^ + O_2_ → Eu^3+^ + O_2_^•−^, (13)

e^−^ + V_0_^++^ → V_0_^+^, (14)

V_0_^+^ + O_2_ → O_2_^•−^, (15)

e^−^ + O_2_^•−^ + 2H^+^ → H_2_O_2_, (16)

e^−^ + H_2_O_2_ → OH^−^ + ^•^OH, (17)

Simultaneously, the VB photogenerated holes undergo trapping with the surface group of hydroxyls, reacting with ionized oxygen defects or reacting with adsorbed water (H_2_O_2_) through transferring of interfacial charges, creating a highly radical reactive hydroxyl (^•^OH) [41,42,43]:

h^+^ + (H_2_O → H^+^ + OH^−^) → H^+^ + ^•^OH, (18)

## 3. Results and Discussions

### 3.1. X-ray Diffraction (XRD) Pattern

**Figure 1:**
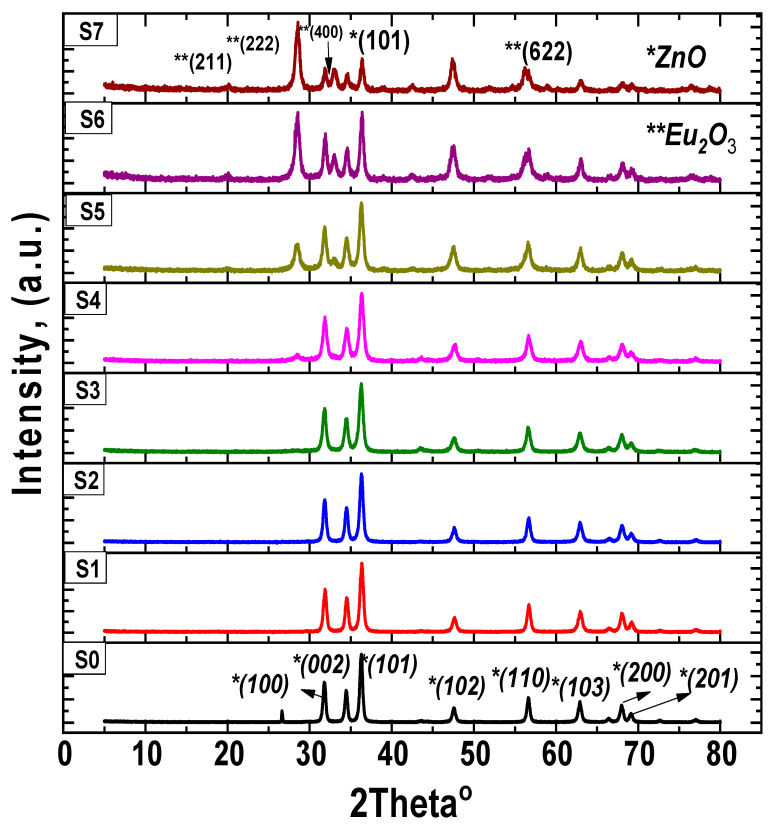
XRD patterns of the prepared Eu_2_O_3_-ZnO nanostructures at different concentrations. * is pure ZnO phase and ** is for Eu_2_O_3_ phase.

Figure 1 shows the measured XRD patterns of the pure and Eu_2_O_3_-doped ZnO nanostructures. It is clear that pure ZnO and Eu_2_O_3_-doped ZnO predominantly illustrated seven XRD peaks and confirmed the wurtzite phase, which is well-matched with JSPDS 01-075-0576. The critical diffraction peaks have the positions at 31.8°, 34.5°, 36.4°, 47.7°, 56.7°, 62.9°, 66.4° and 69.05° with the indices of (1 0 0), (0 0 2), (1 0 1), (1 0 2), (1 1 0), (1 0 3), (2 0 0), and (2 0 1) respectively. The observed diffraction peaks for pure ZnO nanoparticles outstandingly matched the data reported by Aydn et al. [29] and were similar to the reported data by Wang, R. H., et al. [30]. In the case of doping samples, the Eu_2_O_3_-ZnO phase does not appear at low doping, as shown in Table 2 and Figure 1, but does occur as the dopant concentration increases in the pure ZnO matrix. The reason could be explained by dislocations which are crystal flaws in one section of the crystal that cause the lattice to misregister as compared with another part. Further, all the calculated structural parameters of pure and doped samples are depicted in Table 2. 

Fascinating features can be seen in the XRD patterns of the Eu_2_O_3_-ZnO nanostructures with the phase (JSPDS 00-034-0392) with the indices of (2 2 2), (2 1 1), (4 0 0), and (6 2 2). The prominent peaks corresponded to (1 0 0) and (1 0 1) planes that have been detected in all prepared nanomaterials. A slight shift of the (0 0 2) plane towards a value to the left at a higher concentration of doping samples than undoped ZnO material reveals that the content of europium (Eu) dopant significantly affects the crystalline structure of pure material. It is also observed from the XRD graphs that there is a formation of the Eu_2_O_3_ phase at higher concentrations, which suggests the effect of doping into the pure matrix. Furthermore, the mean crystallite size values of all prepared ZnO-doped Eu_2_O_3_ nanocomposites are diverse with variations in doping, in the range of 13–42 nm. In this current study, the attained grain size was in agreement with the published results for silver-ZnO nanomaterial by Wang, R. H. et al. [30]. The Scherrer formula calculated the mean grain size from the XRD spectra, ranging between 20.9 nm and 22.1 nm [30]. The evaluated crystallinity size noticeably increased with an increase in the dopant ratios.

### 3.2. SEM Analysis 

**Figure 2:**
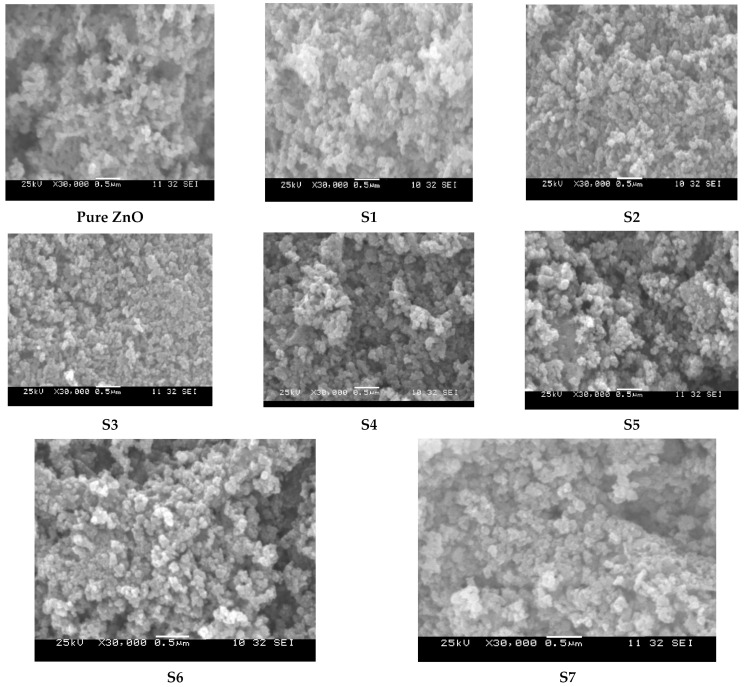
SEM images of pure and Eu_2_O_3_-doped ZnO nanostructured samples, with various europium (Eu) doping ratios (0–5 g).

Scanning electron microscopy (SEM) was used to study the morphology of Eu_2_O_3_-ZnO nanostructures, as illustrated in Figure 2. The structural morphology changes in the pure-ZnO matrix and the europium (Eu)-doped ZnO nanocomposites were directly recognizable. The SEM pictures of Eu_2_O_3_-ZnO nanocomposite powder exhibited a general homogeneous dispersion of spherical nanoparticles. The europium ions strongly formed tiny grains and promoted crystal nucleation, whereas the trapping of Eu grains hindered grain development. The europium ions created grains of small size (ranging between 75 and 89 nm) and encouraged the crystal nucleation rate, while the trapping of europium grains prevented grain development. This trend could be due to the discrepancy in ionic radius between zinc and europium [44]. The form and size of the nanoparticles changed as the amount of Eu doped in them grew, as illustrated in Table 2. The obtained SEM results closely matched the results of Chao, L. C., et al. [45].

### 3.3. Spectroscopic Analysis 

**Figure 3:**
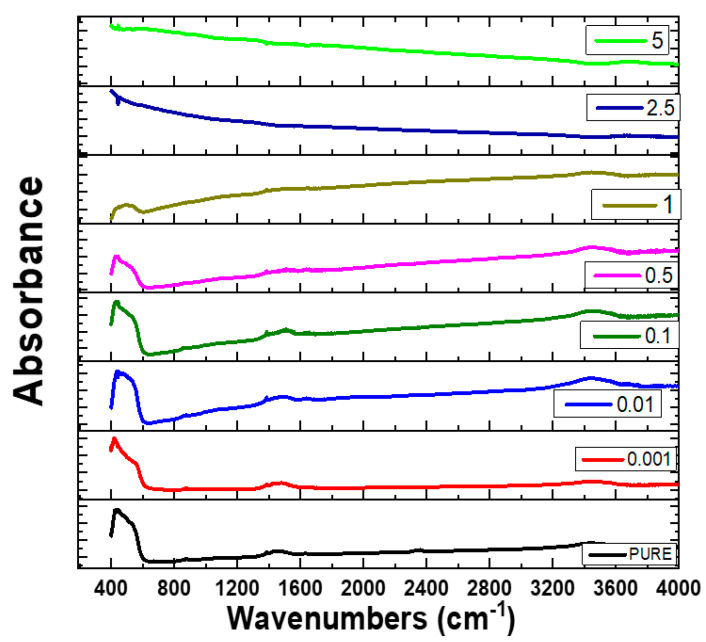
FT-IR spectra of undoped ZnO and Eu_2_O_3_-doped ZnO nanocomposites at different europium (Eu) doping ratios.

Figure 3 shows the FT-IR spectra of the prepared samples in 400–4000 cm^−1^. The undoped ZnO sample’s optical transparency improved marginally compared to Eu doped samples. As the doping ratio for europium (Eu) increased, the transmission of Eu_2_O_3_-ZnO nano samples decreased, possibly due to increased scattering. In the FT-IR spectra, the highly intense broad absorption peak at around 438 cm^−1^ of the wavenumber was credited to the stretching vibrations in the pure ZnO matrix [46]. In addition, a wide absorbance band at about 3450 cm^−1^ characterized the O–H group’s stretching vibrations, while the small absorption peak at around 1635 cm^−1^ corresponded to the bending vibrations of the interlayered molecule. The surface caused the impurity bands of different higher wavenumber adsorbed organic matrices either in the synthesis procedure or the characterization. The impurity effects were extremely predominant for Eu nanoparticles because of the large ratio of surface to volume; however, those impurity bands gradually disappeared in the bulk materials [46]. As a result, the most excellent Eu_2_O_3_ doping ratios (i.e., 5 g) exhibit the highest absorbance, consistent with the structural morphology results from the XRD and SEM investigations. Furthermore, the observed effects were similar to those with the developments of Nd-doped ZnO nanocomposites reported by Chauhan et al. [46].

### 3.4. Optical Characterizations

#### 3.4.1. Optical Diffused Reflectance (ODR) and Absorption Index

**Figure 4:**
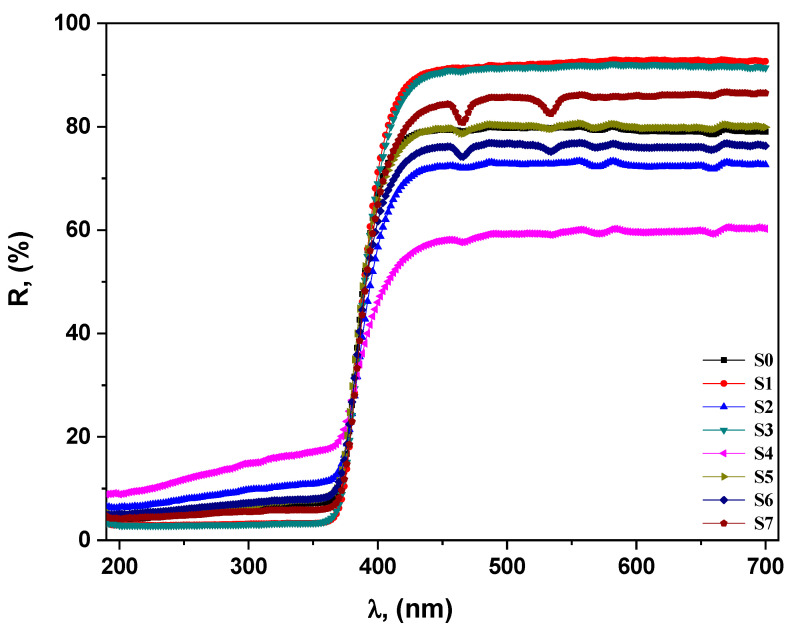
Graph of the optical diffused reflectance (ODR) of all nanostructured Eu_2_O_3_-ZnO samples with different doping ratios of europium versus light wavelength.

**Figure 5:**
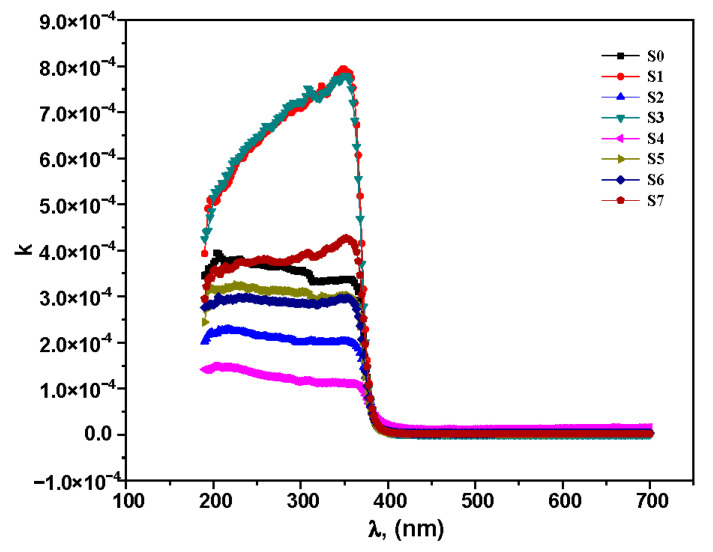
Graph the absorption coefficient (k) of the prepared Eu_2_O_3_-ZnO nanocomposites as a function of light wavelength between 200 nm and 700 nm.

Diffuse reflectance (DR) measurement is a well-known technique for obtaining information on the absorption properties of nanostructured materials. Figure 4 and Figure 5 show the optical diffused reflectance (ODR) and absorption index (*k*) of the Eu_2_O_3_-doped ZnO nanocomposites from 200 nm to 700 nm. From Figure 4, it can be observed that, in the range from 200 nm to 370 nm, no change in ODR data was monitored. However, a substantial increase in the ODR was seen from 370 nm to 410 nm, producing the optical bandgap. Within the wavelength range from 410 to 700 nm, the diffuse optical reflectance (ODR) spectra showed virtually straight curves with few variations.

The calculated ODR agreed with the reported optical results reported by Chaudhary et al. [23]. The optical properties showed that the absorption edges shifted toward the visible region [23]. The optical investigation of sol-gel prepared Nd-doped ZnO nanoparticles reported by Chao, L. C., et al. also demonstrated durable emission of band edge placed at the wavelength of 380 nm, where the defect emission was related to the deep level at about 620 nm wavelength [47]. In conclusion, the absorbed light generates a bandgap of optical absorption via the deliberate samples, which can be explained due to non-absorbent surface area and the predisposition of Eu_2_O_3_-ZnO nanocomposite materials to incident light [48,49]. Figure 5 shows a slightly small absorption index (*k*) of the examined Eu_2_O_3_-doped ZnO nano-samples, ranging between 1 × 10^−4^ and 8 × 10^−4^. The most outstanding absorption values are at a light wavelength of roughly 380 nm. The light will cause electrons to move from their ground state orbitals to higher-energy, excited state or antibonding orbitals. If the molecule has transitions in the ultraviolet (UV) or visible ranges of the electromagnetic spectrum, ultraviolet-visible spectroscopy can be used to determine the electronic transitions. Many molecular electronic transitions exist, i.e., σ → σ∗, π → π*, n → σ∗, n → π∗. Especially in the region of 200–800 nm, the three responsible transitions, i.e., π → π*, n → σ∗, n → π∗, [50,51]. 

#### 3.4.2. Optical Energy Bandgaps

**Figure 6:**
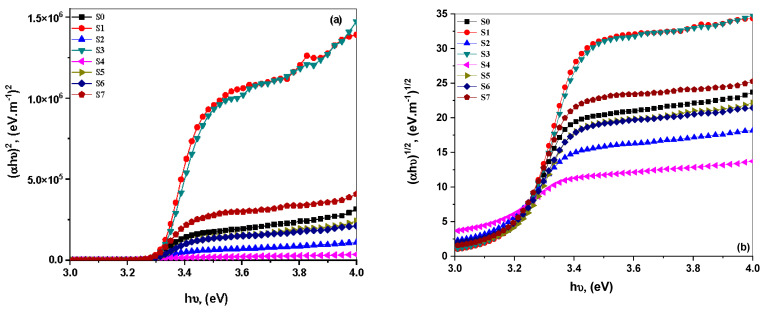
(**a**,**b**) Illustration of the relation between the optical allowed direct (*αhυ*)^2^ and indirect (*αhυ*)^1/2^ transitions of the prepared Eu_2_O_3_-doped ZnO nanocomposites versus the photon energy (*hυ*).

Figure 6a,b show the (*αhυ*)^1/2^ and (*αhυ*)^2^ versus the photon energy (*hυ*) of the Eu_2_O_3_-ZnO samples. On the one hand, the calculated values of direct optical bandgap range from 3.31 eV to 3.24 eV. On the other hand, the indirect optical allowed optical transition values range from 3.23 eV to 3.14 eV. The evaluated optical bandgaps for the synthesized Eu_2_O_3_-doped ZnO nanoparticles are presented in Table 3. The present samples’ direct and indirect bandgap values agree with the recently reported Fe-ZnO nanostructures by Aydın et al. [29] and Chauhan et al. [45]. The decreasing bandgap in the samples could be explained due to the new generation of energy state between the conduction and the valence levels.

### 3.5. Photocatalytic Activity

#### 3.5.1. Photodegradation of Organic Compounds under Investigation Using Prepared Samples

**Figure 7:**
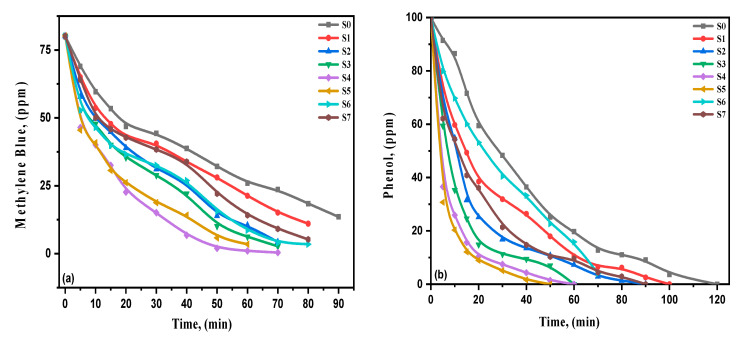
(**a**,**b**) Concentration versus time for photodegradation using Eu_2_O_3_-ZnO of: (**a**) MB; (**b**) phenol.

The photocatalytic activities of pure and Eu_2_O_3_-doped ZnO were examined under visible irradiation using methylene blue as a colored dye and phenol as a colorless organic compound. The absorption spectra of the aqueous MB and Ph solution at various time intervals at irradiation were recorded. The photodegradation was estimated from the reduction in the maximum intensity of the absorption peak. Photocatalytic degradation of MB and Ph is distinguished from the concentration plot of MB or Ph versus irradiation time (min). Figure 7a,b shows the decrease in the MB solution and Ph solution concentrations at various illumination times for pure and Eu_2_O_3_-doped ZnO samples. 

#### 3.5.2. Kinetic Studies of the Photocatalytic Degradation Process

**Figure 8:**
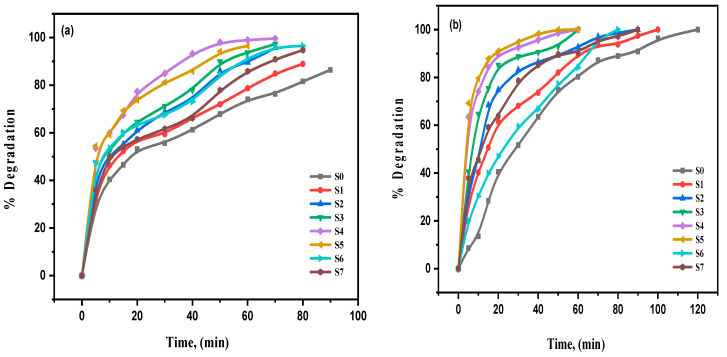
(**a**,**b**) Degradation % using Eu_2_O_3_-doped ZnO nanocomposites of: (**a**) MB; (**b**) phenol.

**Figure 9:**
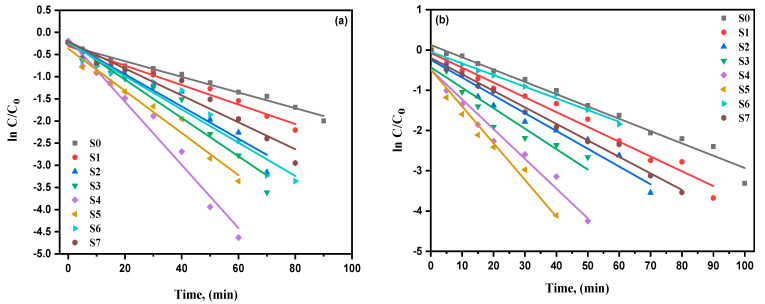
(**a**,**b**) Kinetic data for photodegradation using Eu_2_O_3_-ZnO of: (**a**) MB; (**b**) phenol.

The weight concentrations of Eu increased, and the % of degradation of both MB and Ph increased, as shown in Figure 8a,b. The relationship between *ln (C/C_o_)* and the reaction time is illustrated in Figure 9a,b. The spectrum fitting of photocatalytic degradation of MB or Ph was accomplished to obtain the best straight line. The obtained coefficients of linear regression (R2) were estimated at around one for all the measured samples, indicating that the photodegradation obeyed the pseudo-first-order kinetics. The rate constants for the degradation of MB ranged from 0.017 to 0.07 min^−1^. At the same time, it varied from 0.03 to 0.09 min^−1^ for the phenol degradation for different doping concentrations.

The degradation factors for MB with 0.5% Eu-doped ZnO nanocomposites increase the reaction rate by around four times compared with pure ZnO samples. However, the reaction rate for Ph degradation constants with 1% Eu_2_O_3_-doped ZnO is three times more than that for pure ZnO. Generally, the performance of photocatalyst activities was well-defined through the recombination rate delay of photo-created electron-hole pairs. Consequently, the correct mole fraction of Eu^3+^ ions-doped ZnO nanostructures would enhance the photocatalytic activities due to increased electron-hole lifetime.

Table 4 summarizes the first-order rate coefficient (*k*) values extracted from the linear graphs. The defined k values for pure ZnO and Eu_2_O_3_-doped nanostructures indicated increased rate parameters with increasing Eu dopants. Therefore, the improvement in the Eu-ZnO photocatalytic activities could increase the concentration of Eu nanoparticles, as confirmed through absorption and XRD spectra. Additionally, doping Eu NPs could present in the ZnO lattice both point defects and trapping sites, which results in increased separation among electron-hole pairs, thus, increasing the ZnO photocatalytic efficiency [17]. Moreover, a photo-created electron in the conduction band could be recognized via Eu^+3^ ions. Then, the generated Eu^+2^ could be related to the dissolved O_2_ and produce the radical anion of superoxide O_2_^−^ [22].

#### 3.5.3. Proposed Mechanism of Photodegradation of MB and Ph

**Scheme 1:**
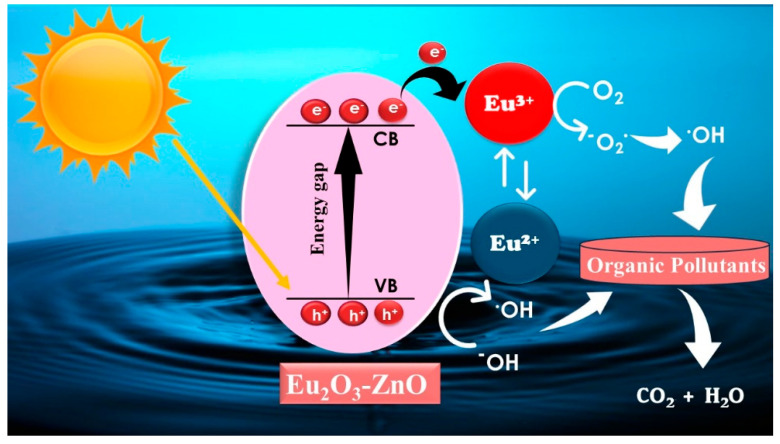
Degradation mechanism for MB and phenol using Eu_2_O_3_-ZnO nanocomposites.

In the literature, investigations of the photocatalytic mechanism of semiconductor materials and the effects of rare earth ions have been extremely recognized [61,62,63,64]. In this study, the VB electrons absorb energy from visible light to irradiate ZnO. Then, those electrons are excited to the conduction band (CB), creating electron-hole pairs (e^−^/h^+^). The reactions of oxidation are initiated from those (e^−^/h^+^) pairs as follows: The dissolved oxygen is reduced to an anion of superoxide radical (O_2_^−^), and the H_2_O molecule is oxidized to (OH) radicals. Here, h^+^, O_2_^−^, and^.^OH, radicals are very significant in contributing to the organic dye’s decomposition to CO_2_ and H_2_O. Even though one challenging factor in an investigation of photocatalytic efficiency is the recombination of the created (e^−^/h^+^) pairs [64]. The degradation mechanism of Eu_2_O_3_-ZnO photocatalytic is illustrated in Scheme 1.

The 0.5 g of Eu_2_O_3_-doped ZnO (S4) displayed 100% MB removal in 80 min. Furthermore, 1 g Eu-doped ZnO degraded 100% phenol in 60 min. The more considerable photocatalytic activity of Eu_2_O_3_-doped ZnO nanocomposites is primarily due to electron movement among the valence bands of Eu, Eu^2+^, and Eu^3+^ [65]. Since europium (Eu) can have two and three valence electrons, Eu^3+^ and Eu^2+^ could trap those electrons and give them oxygen to form radical ions of superoxide (O_2_^−^). It was conveyed that the potential energy of Eu (Eu^3+^/Eu^2+^) reduction is negative about -0.35 eV further than E (O_2_^−^) with +0.12 eV [66]. Therefore, the oxygen absorption on the Eu_2_O_3_-doped ZnO surface could be decreased to reach O_2_^−^. As seen in Scheme 1, the capturing and transferring of electrons increases the Eu_2_O_3_-doped ZnO photocatalytic activities, which results in the effective separation of electron-hole pairs. 

Thus, rapid exciton recombination could enormously reduce the efficiency of the Eu_2_O_3_-ZnO photocatalytic activity. In the case of Eu^3+^ dopants, the electrons are trapped in the energetically favorable Eu^3+^ ions, as in Equation (13). As a result, electrons are inhibited from recombination with holes to generate superoxide radicals (O_2_^•−^), as seen in Equation (14) [67]. Equation (15) illustrates that the transferring of electrons to ionize oxygen vacancies (V_0_^++^) leads to the reaction by adsorbate oxygen (O_2_) to produce radicals of superoxide (O_2_^•^^−^) [33]. As ZnO nanostructures are doped with Eu^3+^ ions, the larger quantity of the created radicals (O_2_^•^^−^) increases the photocatalytic activity. In the degradation process, organic compounds are initially oxidized via the sequential hole transfer and/or the radical attack of hydroxyl ^•^OH [68]. 

Furthermore, the Eu^3+^ dopants could agree to take the CB electrons of ZnO nanostructures to generate Eu^2+^, which could move one electron for dissolving O_2_ to create radical anions of superoxide for preventing the electron-hole recombination, as demonstrated in Scheme 1 [69,70]. As a result, Eu^3+^ on the ZnO surface could be considered a scavenger of electrons. Additionally, Eu^3+^-ZnO nanostructures have a narrow energy bandgap as compared with undoped ZnO semiconductors, which could cause an increase in the stability of electron-hole pairs and enhance the efficiency of photocatalytic degradation. Nevertheless, Eu^3+^ ions charging above their ideal level could perform as a recombination focus on photogenerated electron-hole pairs due to their interaction, which reduces the efficiency of photocatalytic [67,71]. Jinrui Li et al. reported that Eu doping on ZnO exhibited the highest photocurrent density, which indicated that the electron-hole lifetime at the interface of the catalyst was extended [72].

Similarly, different researchers have reported the electron transfer and involvement of superoxide radicals in photodegradation by Eu ions [73]. Paola Franco et al. showed the photocatalytic activities of manufactured materials investigated in EBT dye photodegradation using UV and visible irradiation [57]. The experimentally obtained results illustrated that the Eu-ZnO structures synthesized using the supercritical antisolvent SAS (Eu-ZnO-SAS) technique could assure the highest photocatalytic efficiency. Finally, Phuruangrat et al. investigated the photocatalytic activity of manufactured ZnO and Eu-ZnO nanostructures using MB degradation in aqueous solutions within UV irradiation [58]. The MB photocatalytic efficiency of 3% Eu-ZnO reached 90.51% in 300 min, more extensive than undoped ZnO semiconductor. Yanqing Zong et al. found that the efficiency of MO photodegradation for undoped ZnO was just 75.7% in 180 min, while the efficiency reached 95.3% for 1.0 mol% dopants Eu-ZnO [60]. Conspicuously, as the Eu dopant concentrations increases, the efficiency of photodegradation initially upsurges and, after that, declines. Balachandran et al. used the hydrothermal process to prepare Eu_2_O_3_-ZnO nanoclusters, where the composite was comprised of 91% ZnO and 9% Eu_2_O_3_ [60]. The synthesized Eu_2_O_3_-ZnO nanostructures displayed the more considerable photocatalytic activity of 99.2% in the Rhodamine B (RhB) photodegradation under sunlight in 75 min under neutral pH. Numerous researchers have considered hydroxyl radicals the most vital active materials for impurity degradation. The previous comparison proved the influences of the preparation method, weight concentrations of Eu, and conditions of photocatalytic procedure on the photocatalytic activities of prepared Eu_2_O_3_-doped ZnO materials.

In this present work, 0.5 g Eu-ZnO (0.05 Eu/Zn molar%) nanocomposites displayed the highest MB photocatalytic degradation in 80 min of visible irradiation to reach 100% degradation. However, 1 g Eu-doped ZnO indicated 100% phenol degradation after 60 min. This behavior may be attributed to the oxidation procedure resulting from the electron injection into ZnO NPs, with just one exciting organic compound level. 

#### 3.5.4. Detection of Active Species Responsible for the Photocatalytic Degradation Process

**Figure 10:**
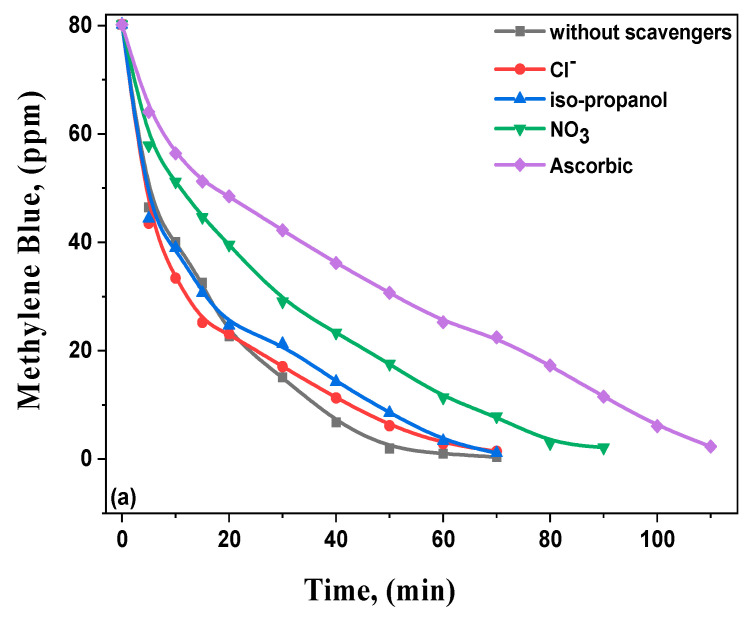

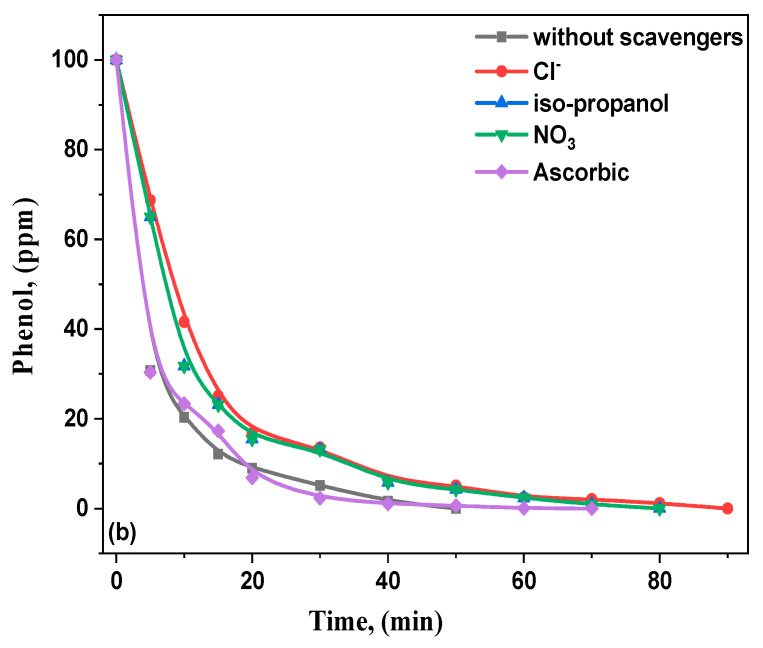
(**a**,**b**) Trapping agent data for photodegradation using Eu_2_O_3_-doped ZnO of: (**a**) MB; (**b**) phenol.

The rare-earth dopants can enhance photocatalytic activities to a substantial degree via the mechanism of charge separation [74]. This significant recombination can be decreased via metal dopant, which professionally traps the charge carriers and increases their lifetime. In this case, Eu^3+^ behaves as traps for electrons, Eu^3+^ ions in the material take the CB electrons, condensed to get Eu^3+^. These produced Eu^3+^ can move those electrons toward adsorbed O_2_^−^ molecules, thus, creating O_2_^−^ radicals. Throughout this procedure, Eu^3+^ would be oxidized as Eu^2+^. Both trapping and the releasing process of electrons by Eu^3+^ significantly decrease the recombination percentage of e^−^-h^+^ pairs [59]. The effects of such scavenging agents have been studied to clarify the main reactive types (photogenerated electrons and holes, superoxide/OH radicals) in the photodegradation of MB and phenol molecules shown in Figure 10a,b. As a hole scavenger, the degradation of 200 mM sodium chloride substantially reduces the catalyst activity against photodegradation of MB and Ph.

The photogenerated holes can react and emit chlorine radicals with the chloride anion, which can respond and form radicals of dichloride anions (Cl_2_) [61]. To produce chlorine and chloride-free anions, two radicals of dichloride anion can efficiently react with each other. To generate radicals of chloro and hydroxyl, the reaction between radicals of dichloride anion and water molecules is also accomplished to get ClOH**^•^**^−^. Chloride has been referred to as a scavenger of OH radicals. IPA can also scavenge a moderately slower rate of ^.^H radicals than OH radicals. OH can be effectively captured by nitrate ions, while **^•^**H and photogenerated electrons can be scavenged to a lesser degree [59]. Ascorbic acid (H_2_A) showed the most extreme decline in MB and Ph degradation as an O_2_^−^ scavenger. The reaction between H_2_A and superoxide radicals could be completed under acidic environments to create ascorbate radicals and H_2_O_2_ with a second-order response. The resulting ascorbate was a relatively non-reactive reaction that decomposed into H_2_A and dehydroascorbic acid through a disproportionate reaction [75].

As confirmed before, the following pattern was found to reduce the photocatalytic behavior of the studied composites in the existence of trapping agents. This pattern, AA > IPA > chloride > nitrate, suggests that the highly significant positions were in MB and phenol photodegradation using the prepared materials, superoxide radicals, and then OH, h^+^ and e^−^. The arrived photons will excite both semiconductors based on these findings, resulting in e^−^/h^+^ pair output in both semiconductors. Degradation in the presence of scavengers reveals the prominent role of superoxide radicals generated by the longer lifetime of electrons caused by the presence of Eu ions. As seen in the scavenger test results, superoxide and holes were the main reactive species that caused the degradation of MB and Ph. In addition, the bandgap energy of this coupled oxide is more minor than ZnO, which makes the catalyst widen its spectral absorption range, extending to the visible region. Eu_2_O_3_-ZnO may have intermittent bands, which causes a reduction in bandgap energy, and this improves its visible light absorption. The photogenerated electrons-hole separation over ZnO/Eu_2_O_3_ heterojunctions enhances the Eu_2_O_3_-doped ZnO performance to be larger than either Eu_2_O_3_ or ZnO nanoparticles.

#### 3.5.5. Recycling of the Prepared Samples

**Figure 11:**
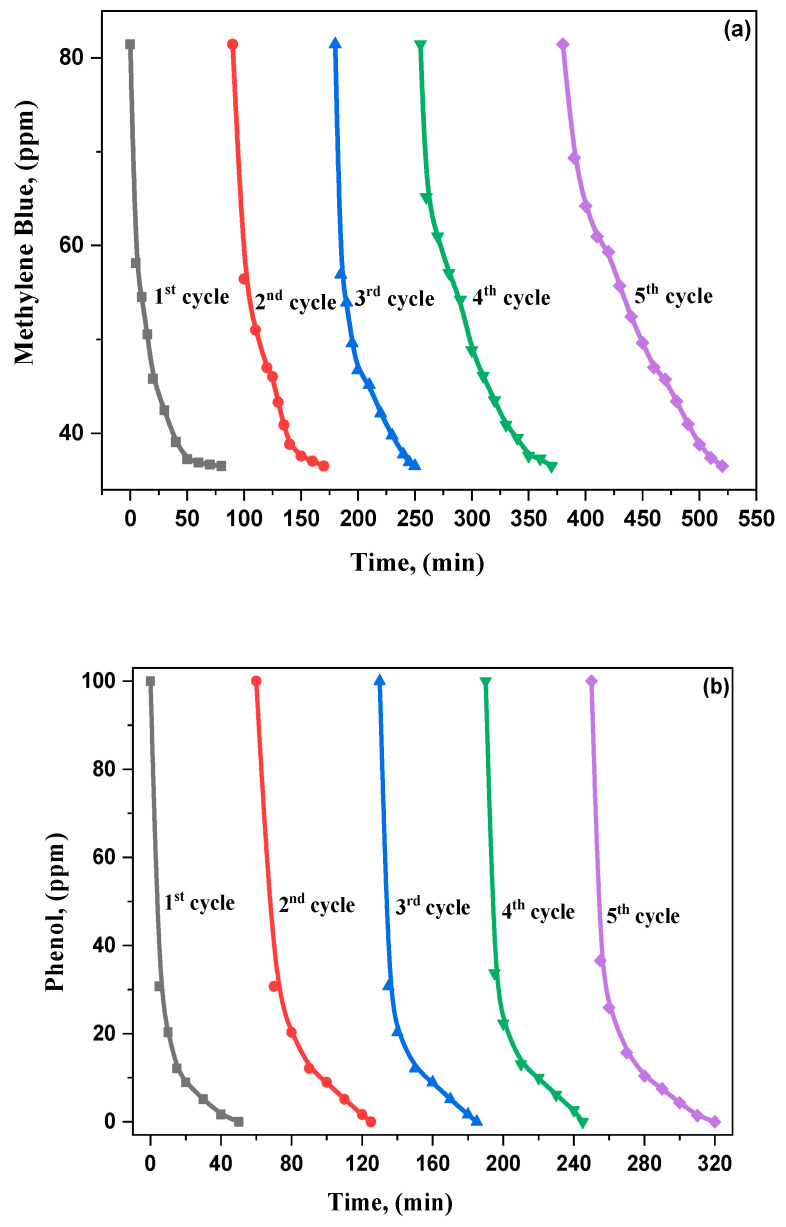
(**a**,**b**) Recycling data for photodegradation in the presence of (**a**) 0.5 g Eu-doped ZnO (S4) of MB; (**b**) 1 g Eu-doped ZnO (S5) of phenol.

The reusability and recyclability of the 0.5 g and 1 g E-ZnO (S4, S5) photocatalysts are correspondingly estimated for MB and phenol photodegradation, respectively. As illustrated in Figure 11a,b, no loss was significantly presented in the efficiency of MB and Ph photodegradation, even after five succeeding series, which confirms the excellent reusability of the photocatalyst. Additionally, recovering the photocatalyst could be efficiently completed using the photoreaction mixtures with just the centrifugal separation approach. The effectively obtained reusability and recyclability of Eu_2_O_3_-doped ZnO photocatalysts could potentially be advantageous in wastewater treatment technological applications.

## 4. Conclusions

In the present work, the combustion technique is a suitable, inexpensive, efficient, and environmentally friendly approach to preparing Eu_2_O_3_-doped ZnO (from 0.001 g to 5 g). The SEM studies confirm an increase in grain size of doped samples compared with pure ZnO nanostructures. The Eu_2_O_3_-ZnO nanocomposites’ optical energy bandgap was between 3.31 eV and 3.24 eV, representing the catalyst absorption edge subject to the ZnO structure. This proposed research concludes that the performance of photocatalytic degradation increases with doping concentration and reaches 100% degradation of MB after 80 min for 0.5 g. Similarly, for 1 g Eu, 100% phenol degradation is reached within 60 min under visible irradiation, indicating the excellent and selective photocatalytic behavior of the produced Eu_2_O_3_-doped ZnO nanocomposites towards the degradation of phenols and methylene blue dyes. The primary role of ^.^O_2_^−^ made from the longer lifetime of electrons due to Eu ions was confirmed to have the highest responsibility for degradation via various trapping agents. Furthermore, this catalyst was recyclable for several runs. Therefore, the nanostructures, innovative and multifunctional materials of Eu_2_O_3_-doped ZnO production, are promising candidates for wide-scale technological, environmental, and biomedical applications, such as optoelectronics and photocatalysis.

## Figures and Tables

**Table 1:** Sample code of pure and doped ZnO nanostructures with different Eu_2_O_3_ concentrations.

Nanostructured Samples	Codes	Eu/Zn Molar%
Pure ZnO	S0	-
0.001 g Eu-doped ZnO	S1	0.0001
0.01 g Eu-doped ZnO	S2	0.001
0.1 g Eu-doped ZnO	S3	0.01
0.5 g Eu-doped ZnO	S4	0.05
1 g Eu-doped ZnO	S5	0.09
2.5 g Eu-doped ZnO	S6	0.21
5 g Eu-doped ZnO	S7	0.35

**Table 2:** The calculated grain size, dislocation, and lattice strain values of all nanostructured Eu_2_O_3_-doped ZnO samples were studied from XRD analysis compared with the SEM results obtained.

Samples	Mean Values from XRD			From SEM Analysis
	Grain Size, (nm)	Dislocation Density, (nm)^−2^	Lattice Strain	Grain Sizes, (nm)
**Pure ZnO**				**88.8**
Phase 1 ZnO	40.2	0.001	0.001	
Phase 2 carbon	33.5	0.002	0.001	
**0.001 g Eu-doped ZnO**				**78.5**
Phase 1 ZnO	42.9	0.001	0.001	
Phase 2 Carbon	30.6	0.002	0.001	
**0.01 g Eu-doped ZnO**				
Phase 1 ZnO	37.8	0.001	0.001	
Phase 2 Carbon	13.6	0.005	0.003	
**0.1 g Eu-doped ZnO**				**75.1**
Phase 1 ZnO	25.3	0.003	0.002	
Phase 2 Carbon	13.7	0.007	0.003	
**0.5 g Eu-doped ZnO**				**75.3**
Phase 1 ZnO	23.9	0.003	0.002	
Phase 2 Eu_2_O_3_	18.3	0.003	0.002	
**1 g Eu-doped ZnO**				**86.7**
Phase 1 ZnO	21.9	0.003	0.002	
Phase 2 Eu_2_O_3_	15.1	0.004	0.002	
**2.5 g Eu-doped ZnO**				**82.5**
Phase 1 ZnO	22.1	0.003	0.002	
Phase 2 Eu_2_O_3_	18.2	0.004	0.002	
**5 g Eu-doped ZnO**				**83.8**
Phase 1 ZnO	24.9	0.002	0.002	
Phase 2 Eu_2_O_3_	18.5	0.003	0.002	

**Table 3:** The calculated optical energy bandgaps (both direct and indirect transitions) of Eu_2_O_3_-doped ZnO nanoparticles.

Samples	*E_g_*_1_(d), eV	*E_g_*_1_(ind), eV
S0	3.28	3.20
S1	3.27	3.19
S2	3.25	3.17
S3	3.29	3.21
S4	3.24	3.14
S5	3.31	3.23
S6	3.30	3.22
S7	3.26	3.18

**Table 4:** Reaction rate constants of MB and Ph degradation in all prepared samples.

Photocatalyst	Doping %	Method of Preparation	Organic Solution	Catalyst Load (g/L)	Irradiation Time	Energy Source	% Degradation	Ref.
**Eu-doped ZnO**	2%	Co-precipitation	Phenol	0.1	30 min	Sun light	97.3%	[51]
**Eu-doped ZnO**	5%	Combustion method	MB	0.05	150 min	UV-light	100%	[52]
**Eu-doped ZnO**	3%	Co-precipitation	RhB	0.02	50 min	UV-light	100%	[53]
**Eu-doped ZnO**	1.62%	Co-precipitation	MB	0.024	80 min	Halogen lamp	99.3%	[54]
**Eu-doped ZnO**	1%	Co-precipitation in water	MB	0.01	150 min	Osram Vitalux lamp	90%	[55]
**Eu-doped ZnO**	3%	Co-precipitation in methanol	MO	0.01	150 min	Osram Vitalux lamp	62%	[55]
**Eu-doped ZnO**	0.46%	Co-precipitation	EBT	3	180 min	UV-light	-	[56]
**Eu-doped ZnO**	3%	Supercritical antisolvent process	EBT	3	240 min	UV-light	-	[57]
**Eu-doped ZnO**	3%	Co-precipitation	MB	0.15	300 min	UV-light	90.5%	[58]
**Eu-doped ZnO**	1%	Co-precipitation	MO	0.1	180 min	UV-light	95.3%	[59]
**Eu-doped ZnO**	9%	Hydrothermal	RhB	0.1	75 min	Visible light	99.2%	[60]
**Eu- doped ZnO**	0.5%	Combustion	MB	0.1	80 min	Visible light	100%	Present work
**Eu-doped ZnO**	1%	Combustion	Phenol	0.1	60 min	Visible light	100%	Present work
